# Triglyceride Mobilization from Lipid Droplets Sustains the Anti-Steatotic Action of Iodothyronines in Cultured Rat Hepatocytes

**DOI:** 10.3389/fphys.2015.00418

**Published:** 2016-01-12

**Authors:** Elena Grasselli, Adriana Voci, Ilaria Demori, Giulia Vecchione, Andrea D. Compalati, Gabriella Gallo, Fernando Goglia, Rita De Matteis, Elena Silvestri, Laura Vergani

**Affiliations:** ^1^Dipartimento di Scienze della Terra, dell'Ambiente e della Vita, Università di GenovaGenova, Italia; ^2^Istituto Nazionale Biostrutture e BiosistemiRoma, Italia; ^3^Dipartimento di Scienze e Tecnologie, Università del SannioBenevento, Italia; ^4^Dipartimento di Scienze Biomolecolari, Università di UrbinoUrbino, Italia

**Keywords:** iodothyronines, primary cultured rat hepatocytes, *in vitro* steatosis, lipid lowering action, adipose triglyceride lipase, lipid droplet, mitochondrial fatty acid oxidation

## Abstract

Adipose tissue, dietary lipids and *de novo* lipogenesis are sources of hepatic free fatty acids (FFAs) that are stored in lipid droplets (LDs) as triacylglycerols (TAGs). Destiny of TAGs stored in LDs is determined by LD proteomic equipment. When adipose triglyceride lipase (ATGL) localizes at LD surface the lipid mobilization is stimulated. In this work, an *in vitro* model of cultured rat hepatocytes mimicking a mild steatosis condition was used to investigate the direct lipid-lowering action of iodothyronines, by focusing, in particular, on LD-associated proteins, FFA oxidation and lipid secretion. Our results demonstrate that in “steatotic” hepatocytes iodothyronines reduced the lipid excess through the recruitment of ATGL on LD surface, and the modulation of the LD-associated proteins Rab18 and TIP47. As an effect of ATGL recruitment, iodothyronines stimulated the lipid mobilization from LDs then followed by the up-regulation of carnitine-palmitoyl-transferase (CPT1) expression and the stimulation of cytochrome-c oxidase (COX) activity that seems to indicate a stimulation of mitochondrial function. The lipid lowering action of iodothyronines did not depend on increased TAG secretion. On the basis of our data, ATGL could be indicated as an early mediator of the lipid-lowering action of iodothyronines able to channel hydrolyzed FFAs toward mitochondrial beta-oxidation rather than secretion.

## Introduction

Hepatic lipid accumulation results from both an increased uptake of circulating free fatty acids (FFAs) rising from adipose tissue or dietary lipids, and/or *de novo* lipogenesis. Excess lipid accumulation leads to hepatic steatosis which is characterized by the accumulation of triacylglycerols (TAGs) in cytosolic lipid droplets (LDs) (Khor et al., 2013). LD accumulation maintains low intracellular level of free fatty acids (FFAs) to avoid their toxic effects on cellular physiology. LDs consist of a core of neutral lipids (mainly TAGs) that is bounded by a monolayer of phospholipids and LD coat proteins. In the past, LDs were considered passive fat depots, but now they are recognized as dynamic organelles at the hub of lipid and energy metabolism (Thiam et al., 2013). The dynamicity of LDs is documented by changes in the expression of LD proteome that reflect the metabolic status of the cell and contribute in regulating lipid metabolism and, ultimately, lipid homeostasis (Pol et al., 2014). The most documented group of LD-associated proteins is the perilipin (PLIN) family (Kimmel et al., 2010), which comprises: perilipin (Plin1), adipophilin/ADRP (adipose differentiation related protein; Plin2), TIP47 (tail-interacting protein of 47 kDa; Plin3), S3-12 (Plin4) and OXPAT (oxidative tissue-enriched PAT protein; Plin5; Bickel et al., 2009). Rab proteins, as key regulators of membrane trafficking, are involved in LD interactions (Murphy et al., 2009). Among the Rab family, LD-associated Rab18 plays a crucial role in the vesicle trafficking between LDs and other organelles (such as mitochondria, peroxisomes, endoplasmic reticulum (ER)) and regulates LD quantity and diameter by controlling fusion/fission processes (Stenmark, 2009; Kiss and Nilsson, 2014). It is now accepted that storage and release of FFAs from LDs result from variations in the expression and/or activity of PLIN proteins and associated lipases. According to metabolic needs, the hepatic lipases dissociate non-esterified fatty acids (NEFAs) from TAGs for oxidation, or alternatively, for re-esterification in the ER, where TAGs are packaged into apolipoprotein-B-containing VLDL (very low density lipoprotein) for secretion (Yao et al., 2013). Adipose triglyceride lipase (ATGL) is now universally recognized as the first and key enzyme that catalyzes the initial step in TAG hydrolysis in both adipose and non-adipose tissues (Watt and Steinberg, 2008). ATGL localizes at LD surface where it plays an important role in LD degradation and lipid mobilization (Smirnova et al., 2006). NEFAs resulting from TAG hydrolysis are principally oxidized through mitochondrial and peroxisomal beta-oxidation. Increases in fatty acid oxidation lead to the stimulation of the respiratory chain, that is inevitably coupled with reactive oxygen species (ROS) production. However, mammalian cells are well equipped with many enzymatic (such as catalase and superoxide dismutase) and non-enzymatic (i.e., glutathione-GSH and metallothioneins-MTs) antioxidant systems able to scavenge ROS.

Thyroid hormones (THs), thyroxine (T_4_) and 3,3′,5-L-triiodothyronine (T_3_), are widely known as key modulators of energy balance and lipid metabolism. In the last decades, even 3,5-diiodo-L-thyronine (T_2_) has been shown to exert thyromimetic actions. *In vivo* and *in vitro* models of hepatic steatosis have been used to demonstrate that T_2_ is able to both prevent (Lanni et al., 2005; Grasselli et al., 2008, 2011a,b, 2012) and reduce (Mollica et al., 2009) fat accumulation. Moreover, T_2_ enhances mitochondrial respiration in both normothyroid (Lombardi et al., 1998) and hypothyroid (Mangiullo et al., 2010; Cavallo et al., 2013) rats by stimulating NEFA oxidation and bioenergetics parameters. However, the hepatic targets of the lipid-lowering effects of T_2_ are largely unknown.

In this study, we used an *in vitro* model of hepatic steatosis to investigate the mechanisms underlying the lipid-lowering action of iodothyronines (T_3_ and T_2_). Our results indicate that both T_3_ and T_2_ induce ATGL recruitment at LD surface and changes in the expression pattern of LD-associated proteins, as well as up-regulation of CPT1 and stimulation of COX activity thus suggesting a stimulation of the mitochondrial function.

## Materials and methods

### Rat hepatocyte culture

Hepatocytes were isolated from adult male Wistar rats (Harlan-Italy, S. Pietro al Natisone, Italy) and cultured as previously described (Fugassa et al., 1983). Animal maintenance and treatment were carried out according to the guidelines of the European Community Council for animal care and use. Twenty-four hours after plating, hepatocytes were incubated with a mixture of NEFAs (oleate/palmitate 2:1 molar ratio, final concentration 1.5 mM) for 24 h (Grasselli et al., 2011a). Control hepatocytes were incubated in the medium without addition of NEFAs. Afterwards, medium was replaced by fresh D-MEM containing T_2_ or T_3_ at two different concentrations (10^−6^M and 10^−5^M) and the cells were incubated for 24 h. As controls (C), hepatocytes were cultured with addition of the vehicle alone. At the end of treatments, hepatocytes were collected and stored at −80°C until use. Cell viability, evaluated by Trypan blue exclusion test, was greater than 90% and it was not affected by treatments. For histological analyses, hepatocytes were cultured and treated directly on collagen-coated glass slides (Falcon, BD, Milano, Italy).

### Histological and ATGL-immunohistochemical staining

Hepatocytes were fixed with 4% buffered formalin. In intact cells, neutral lipids were visualized using the soluble selective dye Oil Red O (ORO) (Koopman et al., 2001). After treatments, slides were washed with potassium phosphate buffer (PBS) and fixed with 4% paraformaldehyde in PBS at 4°C for 1 h, washed in the same buffer and then incubated in 0.3% ORO solution as previously described (Grasselli et al., 2010). ORO-stained cells were then counterstained with haematoxylin and slides were mounted in 10% glycerol in PBS.

ATGL was detected using anti-ATGL antibody (diluted 1:400, cat no. ab85858; Abcam, Cambridge, UK). The immunoreaction was revealed with the avidin-biotin-peroxidase complex (ABC) method (Vector, Burlingame, CA, USA). Preparations were examined using a Nikon light microscope (Nikon Eclipse 80i microscope, Laboratory Imaging, Czech Republic) and ACT-2U image analyzer linked to a Sony equipped with digital camera.

### Lipid quantification and peroxidation

TAG content was quantified using the “Triglycerides liquid” kit (Sentinel, Milan, Italy) that allows to quantify glycerol as a measure of insoluble TAGs extracted with chloroform-methanol (v/v) mixture (Grasselli et al., 2010). A Varian Cary 50 spectrophotometer (Agilent, Milan, Italy) was used for spectrophotometric analysis.

Lipid peroxidation was evaluated by the thiobarbituric acid reactive substances (TBARS) assay using malondialdehyde (MDA; 1,1,3,3-tetramethoxypropane) as a standard (Iguchi et al., 1993).

Values obtained were normalized for the protein content determined by the bicinchoninic acid (BCA) assay using BSA as a standard (Wiechelman et al., 1988). Data are expressed as percent TAGs content relative to controls.

### RNA extraction and real-time quantitative PCR

Total RNA was extracted by using Trizol Reagent (Sigma-Aldrich) according to the manufacturer's instructions. First-strand cDNA was synthesized using 200 RevertAid H-Minus M-MuLV Reverse Transcriptase (Fermentas, Hannover MD, USA) as described elsewhere (Grasselli et al., 2010). Real-time quantitative (qPCR) reactions were performed in quadruplicate in a final volume of 25 μl using 1x SybrGreen PCR Master Mix and were analyzed in 96-well optical reaction by Chromo4 System PCR (Biorad, Monza, Italy) as previously described (Grasselli et al., 2012). Primer pairs for the genes under analysis (Table 1) were designed *ad hoc* starting from the coding sequences of Rattus norvegicus available on the GenBank database (http://www.ncbi.nlm.nih.gov/Genbank/GenbankSearch.html) and synthesized by TibMolBiol custom oligosynthesis service (Genova, Italy). Amplification conditions were as follows: 3 min at 95°C, followed by 5 s at 95°C and 1 min at 60°C or 64°C for 40 cycles. A melting curve of qPCR products (65–94°C) was also performed to ensure the absence of artifacts. The relative quantity of target mRNA was calculated by using the comparative Cq method and was normalized for the expression of GAPDH gene. The normalized expression of the target genes was thus expressed as relative quantity of mRNA (fold induction) with respect to controls (C) (Pfaffl, 2001).

**Table 1:** **Characteristics of the primer pairs used for RT-qPCR analysis**.

**PRIMER NAME**	**Primer sequence (5′ → 3′)**	**Annealing temperature (°C)**	**Product lenght (bp)**	**Accession ID**
GAPDH Fwd	GACCCCTTCATTGACCTCAAC	60	136	DQ403053
GAPDH Rev	CGCTCCTGGAAGATGGTGATGGG			
Rab18 Fwd	GGGACCTTGCAGTTTGCAC	64	134	BC089957
Rab18 Rev	CCCCCCCCTCAAAAAACCCC			
TIP47 Fwd	GGAACTGGTGTCATCAACAG	60	108	NW_047865.1
TIP47 Rev	GGTCACATCCACTGCTCCTG			
ADRP Fwd	CCGAGCGTGGTGACGAGGG	64	148	AAH85861
ADRP Rev	GAGGTCACGGTCCTCACTCCC			
CPT1 Fwd	CCGCTCATGGTCAACAGCA	60	105	NM_031559
CPT1 Rev	CAGCAGTATGGCGTGGATGG			
MT-1 Fwd	CTGCTCCACCGGCGG	60	123	AY341880
MT-1 Rev	GCCCTGGGCACATTTGG			
MT-2 Fwd	TCCTGTGCCACAGATGGATC	60	149	XM_001070713
MT-2 Rev	GTCCGAAGCCTCTTTGCAGA			
ApoB100 Fwd	CGTGGGCTCCAGCATTCTA	60	71	NM_019287.2
ApoB100 Rev	TCACCAGTCATTTCTGCCTTTG			

### Western blot

For ATGL immunodetection, cells were lysed in buffer containing 1% Triton and 1% Natrium deoxycholate. Thirty-five to fifty micrograms of protein extract of each sample were electrophoresed at 70 mA on 12% SDS–polyacrylamide gel (SDS-PAGE) (Laemmli, 1970). After electrophoretic run, the gel was electroblotted onto a nitrocellulose membrane using Towbin buffer (25 mM Tris HCl, 192 mM glycine, 20% methanol; pH 8.3; Towbin et al., 1979). Then, membrane was blocked for 1 h in 5% fat-free milk/PBS pH 7.4 solution. As primary antibodies we used rabbit anti-human ATGL (sc-365278) supplied by Santa Cruz Biotechnology (DBA, Milan, Italy). Membranes were incubated overnight at 4°C with primary antibody in PBS, then washed twice in PBST buffer (0.1 M phosphate buffer, 0.0027 M KCl, 0.137 M NaCl, and 0.1% Tween 20, in PBS pH 7.4) and once in PBS. Then, membranes were incubated with horseradish peroxidase (HRP)-conjugated goat anti-mouse IgG (Sigma-Aldrich, Milan, Italy) or rabbit anti-goat IgG (Biorad) as a secondary antibody in PBST for 1 h at room temperature. Protein molecular weight markers were from Biorad (Plus Protein™ Dual Xtra Standards). As loading controls, membrane was stripped and reprobed with rabbit anti-actin antibody (A2066; Sigma-Aldrich, Milan Italy) diluted 1:500 in 5% milk/PBS. Immune complexes were visualized using an enhanced chemiluminescence western blotting analysis system (Bio-Rad ChemiDoc XRS System). Western blot films were digitized and band optical densities were quantified against the actin band using a computerized imaging system and expressed as Relative Optical Density (ROD, arbitrary units). ROD of each band was expressed as percentage with respect to controls.

### Blue-native page and histochemical staining

Solubilization of mitochondrial membranes by detergents, blue-native (BN) PAGE, staining, and densitometric quantification of oxidative phosphorylation complexes were performed essentially as described elsewhere, with minor modifications (Silvestri et al., 2015). Briefly, cells were suspended in hypotonic buffer (83 mM sucrose, 10 mM MOPS, pH 7.2), homogenized in a tightly fitting glass-Teflon homogenizer, and mixed with 250 mM sucrose, 30 mM MOPS, pH 7.2. After a 15 min centrifugation at 600 × g (4°C), to remove broken cells, the mitochondrial fraction was collected by a 15 min centrifugation at 15,000 × g (4°C). The mitochondria-containing sediment was suspended in 1 M 6-aminohexanoic acid, 50 mM Bis-Tris-HCl, pH 7.0; then the membrane proteins were solubilized by addition of 10% (w/v) dodecylmaltoside (specific for solubilization of individual respiratory chain complexes). Following a 15-min centrifugation at 100,000 × g, Coomassie-G250 dye (5% in 1 M aminohexanoic acid) was added to the supernatant and the sample was applied to a 6–13% gradient polyacrylamide gel for BN-PAGE. Each lane contained 15 μg of mitochondrial protein extract. After the runs, gels were fixed and stained with Coomassie Blue G. The band patterns were scanned using an imaging system (Bio-Rad model GS-800 densitometer). The areas of the bands were expressed as absolute values (arbitrary units). In parallel, another a electrophoretic run was performed and enzymatic colorimetric reaction was carried out essentially as reported by others with minor modifications (Zerbetto et al., 1997). Complex IV (COX) activity was estimated by incubating BN-PAGE gels with 5 mg 3.3 0-diaminobenzidine tetrahydrochloride (DAB) dissolved in 9 mL phosphate buffer (0.05 M, pH 7.4), 1 mL catalase (20 μg/mL), 10 mg cytochrome c, and 750 mg sucrose. The intensity of the reactive bands was normalized relative to the intensity of the corresponding bands detected by Coomassie Blue G staining.

### Statistics

Data on both q-PCR and western blot are means ± S.D. of at least four independent experiments performed in triplicate. Data on enzyme activity are means ± S.D. of at least three independent experiments. Statistical analysis was performed by using ANOVA followed by Bonferroni *post hoc* test.

## Results

### Effects of iodothyronines on ATGL recruitment on LD surface

**Figure 1:**
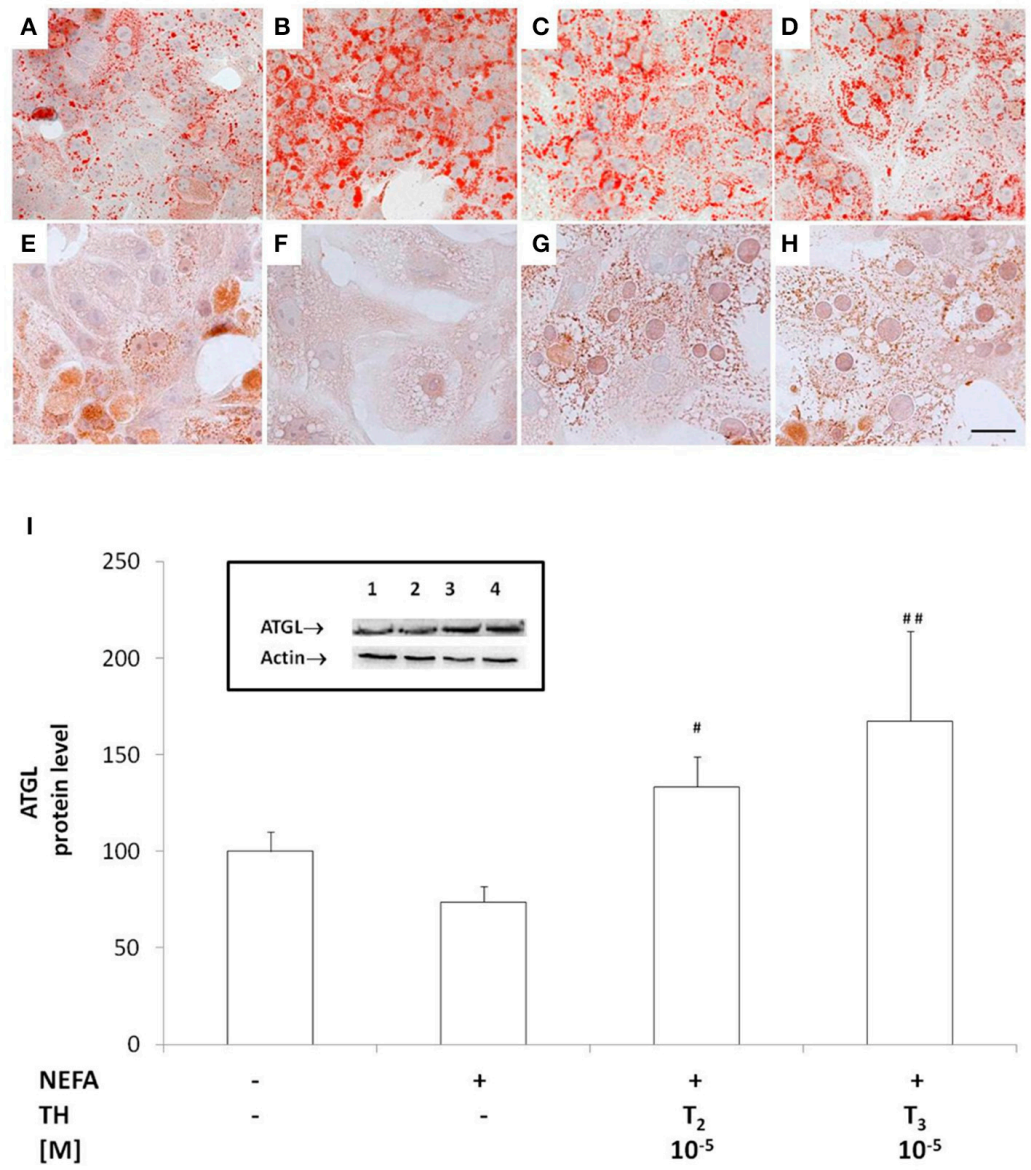
**Effects of iodothyronines on lipid accumulation and ATGL recruitment/expression**. Representative images of rat hepatocytes upon ORO **(A–D)** and ATGL immunohistochemical **(E–H)** staining. The panels report control **(A,E)** and “steatotic” hepatocytes incubated in the absence **(B,F)** or in the presence of T_2_ **(C,G)** or T_3_ **(D,H)** (10^−5^ M) for 24 h. Nuclear staining with haematoxylin is also shown (Bar: 25 μm). Histogram showing ATGL protein level as evaluated by western blot **(I)**. Actin was the protein loading control in SDS-PAGE. In the inset, a representative image of ATGL immune-reactive bands activity is reported (lane1: control, lane2: NEFA, lane3: NEFA+T_2_ 10^−5^ M/24 h, lane4: NEFA+T_3_ 10^−5^ M/24h). Data (mean ± S.D. of at least four independent replicates) are expressed with respect to controls taken as 100. Significant differences are reported (NEFA vs. THs, ^*##*^*p* ≤ 0.01 and ^*#*^*p* ≤ 0.05).

Primary rat hepatocytes exposed for 24 h to a mixture of NEFAs (oleate/palmitate 1.5 mM) mimic a mild steatosis condition that is reversed by treatment with T_2_, or T_3_, for 24 h. ORO staining and microscopic analysis evidenced small (maximum diameter 3.3 μm) and few (maximum 5 LDs/cell) lipid droplets diffused throughout the cytoplasm of control hepatocytes (Figure 1A). In “steatotic” hepatocytes, the excess fat led to an increase in both the number (maximum 15 LDs/cell) and the size (maximum diameter 20 μm) of LDs, with respect to controls (Figure 1B). Treatment of steatotic hepatocytes with both T_2_ and T_3_ was associated with a halving of both LD parameters (maximum diameter was reduced to about 10 μm and maximum number to 7 LDs/cell; Figures 1C,D).

The recruitment of ATGL on LD surface was assessed by immunostaining (Figures 1E–H). While in control hepatocytes, cytosol was punctuated by small ATGL-positive droplets (Figure 1E), in “steatotic” hepatocytes, fat accumulation was accompanied by a reduction in the number of small ATGL-positive droplets, with the appearance of numerous and large ATGL-negative droplets (Figure 1F). The lipid-lowering effect of T_2_ or T_3_ (10^−5^ M) led to the reappearance of numerous small ATGL-positive droplets, similarly to those observed in control cells (Figures 1G,H).

The levels of ATGL protein were quantified by western blot. No increase in ATGL content was observed in “steatotic” hepatocytes with respect to controls (Figure 1I), but treatment of “steatotic” cells with iodothyronines induced a significant increase in ATGL protein levels (about +30% *p* < 0.05 for T_2_ and about +70% *p* < 0.01 for T_3_ with respect to “steatotic” cells).

### Effects of iodothyronines on the expression proteins of LD

**Figure 2:**
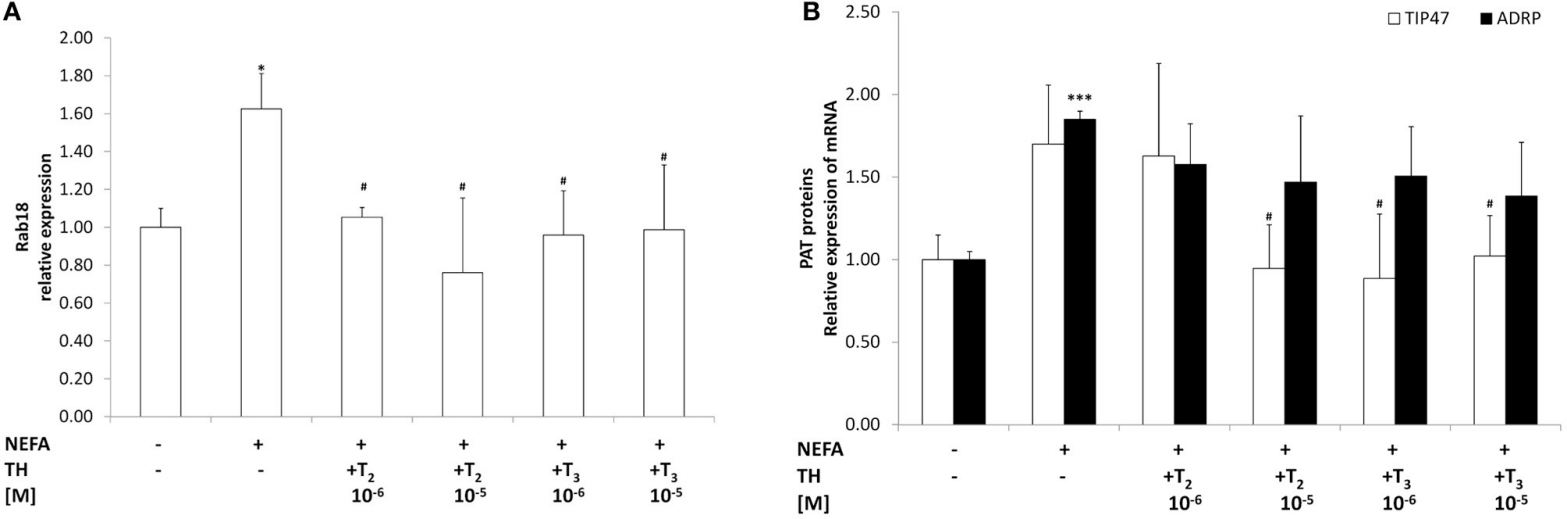
**Effects of iodothyronines on lipid droplet associated proteins**. Relative mRNA expression of Rab18 **(A)** and TIP47(white) and ADRP (black) **(B)** was evaluated by qPCR in control and “steatotic” cells incubated in the absence (NEFA) or in the presence of T_2_ or T_3_ (10^−6^ and 10^−5^ M, 24 h). GAPDH was used as the internal control for quantifying gene expression. Data, expressed with respect to controls, are the mean ± S.D. of at least four experiments in triplicate. Significant differences are denoted by symbols on bars (C vs. NEFA ^***^p ≤ 0.001 and ^*^p ≤ 0.05; NEFA vs. THs ^*#*^*p* ≤ 0.05).

The mRNA expression of the LD-associated proteins Rab18, TIP47, ADRP and OXPAT was assessed by qPCR (Figure 2). In'steatotic' cells, there was a significant up-regulation of Rab18 expression (about 1.60 folds, *p* ≤ 0.05 with respect to controls Figure 2A) that was reduced when “steatotic” hepatocytes were treated with T_2_ (about −35% for 10^−6^M and −50% for 10^−5^M doses, *p* ≤ 0.05 and *p* ≤ 0.01 respectively, compared to “steatotic” cells'). A similar effect was observed with T_3_ (about −40% for both doses, *p* ≤ 0.05 with respect to “steatotic” cells).

A significant up-regulation of ADRP expression was recorded in “steatotic” cells, whereas no changes in TIP47 mRNA expression was observed in “steatotic” cells (Figure 2B). On the other hand, TIP47 expression significantly decreased in “steatotic” hepatocytes treated with the highest dose of T_2_ and both doses of T_3_ (about -45% for T_2_ 10^−5^M; -50% for T_3_ 10^−6^M and −40% for T_3_ 10^−5^M, *p* ≤ 0.05 with respect to “steatotic” cells). No changes in ADRP expression were detected for any of the concentration of T_2_ or T_3_ tested here. On the other hand, neither T_2_ nor T_3_ affected Rab18 and TIP47 expression in control cells (data not shown). We want to underline that no changes in OXPAT expression were measured for all treatments tested here (data not shown).

### Effects of iodothyronines on mitochondrial function

**Figure 3:**
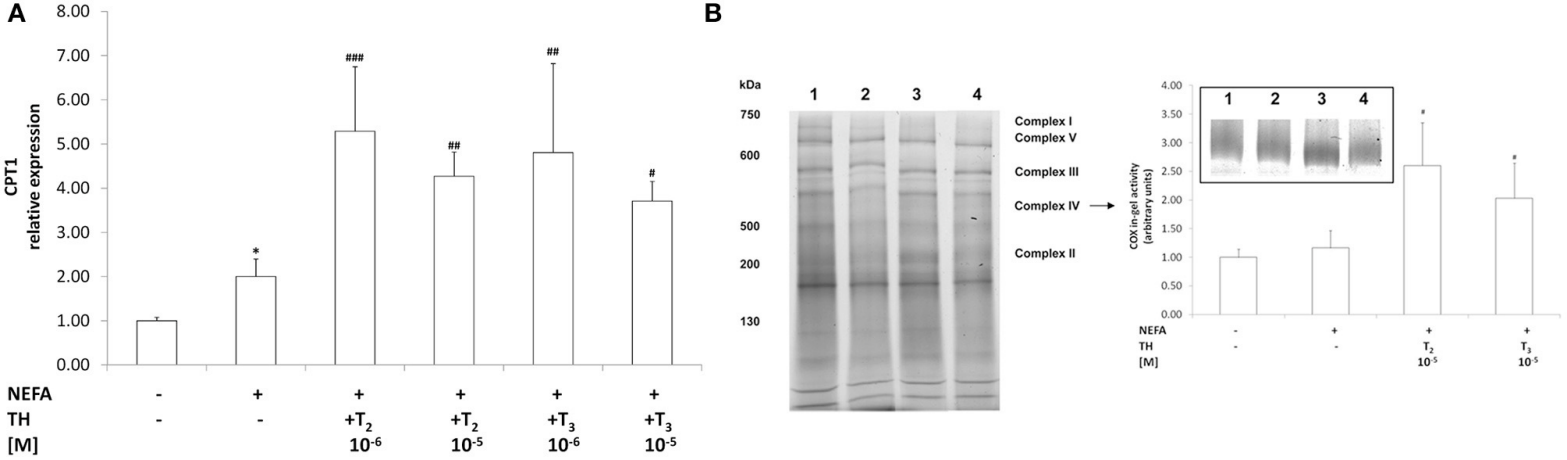
**Effects of iodothyronines on mitochondrial oxidative capacity**. Relative mRNA expression of CPT1 **(A)** was evaluated by qPCR in control and “steatotic” cells incubated in the absence (NEFA) or in the presence of T_2_ or T_3_ (10^−6^ and 10^−5^ M, 24 h). GAPDH was used as the internal control for quantifying gene expression. Data, expressed with respect to controls, are the mean ± S.D. of at least four experiments in triplicate. **(B)** In-gel activity of COX was evaluated by BN-PAGE. Data expressed with respect to controls, are the mean ± S.D. of at least three independent experiments. A representative image of a Coomassie blue stained BN-PAGE gel is reported (on the left). Molecular weights of standard proteins and the relative position of the respiratory complexes are indicated. In the inset, a representative image of COX in-gel activity is also reported (lane1, control; lane2, NEFA; lane3, NEFA+T_2_ 10^−5^ M/24 h; lane4, NEFA+T_3_ 10^−5^ M/24 h). Significant differences are denoted by symbols on bars (C vs. NEFA ^*^p ≤ 0.05; NEFA vs. THs ^*###*^*p* ≤ 0.001; ^*##*^*p* ≤ 0.01; ^*#*^*p* ≤ 0.05).

As an index of mitochondrial oxidation of fatty acids we evaluated the mRNA expression of CPT1, the rate-limiting enzyme step for a major part of beta-oxidation. In “steatotic” hepatocytes, the mRNA levels of CPT1 were up-regulated (about 2.00 fold *p* ≤ 0.05, with respect to controls Figure 3A). CPT1 expression was further increased when “steatotic” hepatocytes were treated with T_2_ (about +165% for 10^−6^M, and +115% for 10^−5^M doses, *p* ≤ 0.001 and *p* ≤ 0.01 respectively, compared to “steatotic” cells). A similar effect was observed with T_3_ (about +140% for 10^−6^M, and +85% for 10^−5^M doses, *p* ≤ 0.01 and *p* ≤ 0.05 respectively, compared to “steatotic” cells). On the other hand, neither T_2_ nor T_3_ affected CPT1 expression in control cells (data not shown).

As an index of mitochondrial respiration, we measured the activity of Complex IV using the enzymatic in-gel activity assay (Figure 3B). No significant change in COX activity was observed in'steatotic' cells with respect to controls (Figure 3B). Treatment of “steatotic” hepatocytes with the highest dose of both T_2_ and T_3_ led to a significant stimulation in COX activity (about +124% for T_2_ 10^−5^M, and +75% for T_3_ 10^−5^M; *p* ≤ 0.05, compared to steatotic cells) On the other hand, neither T_2_ nor T_3_ affected COX activity in control cells (data not shown). In all the experimental conditions, no significant changes were observed as far as it concerns the in-gel activities of both complex I and II.

### Effects of iodothyronines on oxidative stress

**Figure 4:**
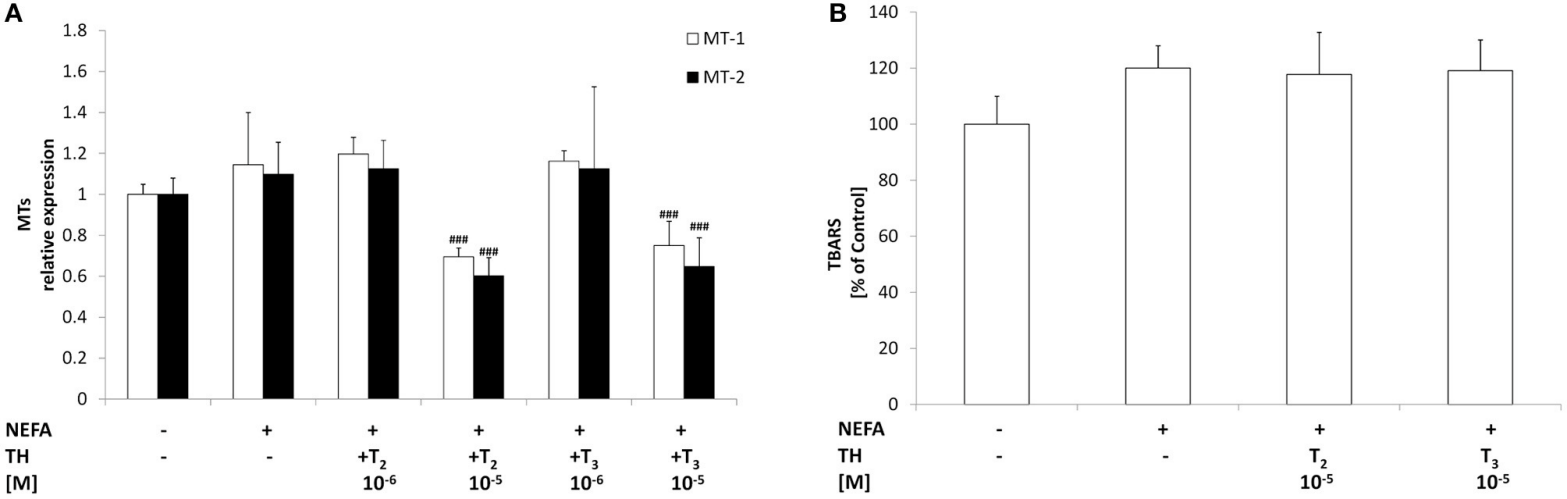
**Effects of iodothyronines on oxidative stress**. Relative mRNA expression of the two hepatic MT isoforms MT1 and MT2 **(A)** was evaluated in control and “steatotic” cells incubated in the absence (NEFA) or in the presence of T_2_ or T_3_ (10^−5^ M, 24 h). GAPDH was used as the internal control for quantifying gene expression. Extent of MDA production **(B)** was measured by TBARS assay. Data, expressed with respect to controls, are the mean ± S.D. of at least four experiments in triplicate. Significant differences are denoted by symbols on bars (NEFA vs. THs ^*###*^*p* ≤ 0.001).

The level of TBARS was assessed as a measure of lipid peroxidation, a classical marker of oxidative stress. No significant changes in TBARS levels were detected as a response to excess lipid accumulation (Figure 4B), thus confirming that our experimental model mimics a mild steatosis condition. Treatment of “steatotic” hepatocytes with both T_2_ and T_3_ did not induce changes in TBARS levels.

Expression of metallothioneins, the main non-enzymatic antioxidants together with glutathione, was determined by qPCR. Lipid accumulation in “steatotic” cells was not associated with significant changes in the expression of both metallothionein isoforms MT-1 and MT-2 (Figure 4A). Treatment of “steatotic” hepatocytes with the highest dose of both T_2_ and T_3_ showed a significant down-regulation of both MT-1 (−40% for T_2_ 10^−5^M and −35% for T_3_ 10^−5^M; *p* ≤ 0.001) and MT-2 (−45% for T_2_ 10^−5^M and −40% for T_3_ 10^−5^M; *p* ≤ 0.001).

Neither T_2_ nor T_3_ affected TBARS levels or MT mRNA expression in control cells (data not shown).

### Effects of iodothyronines on lipid secretion

**Figure 5:**
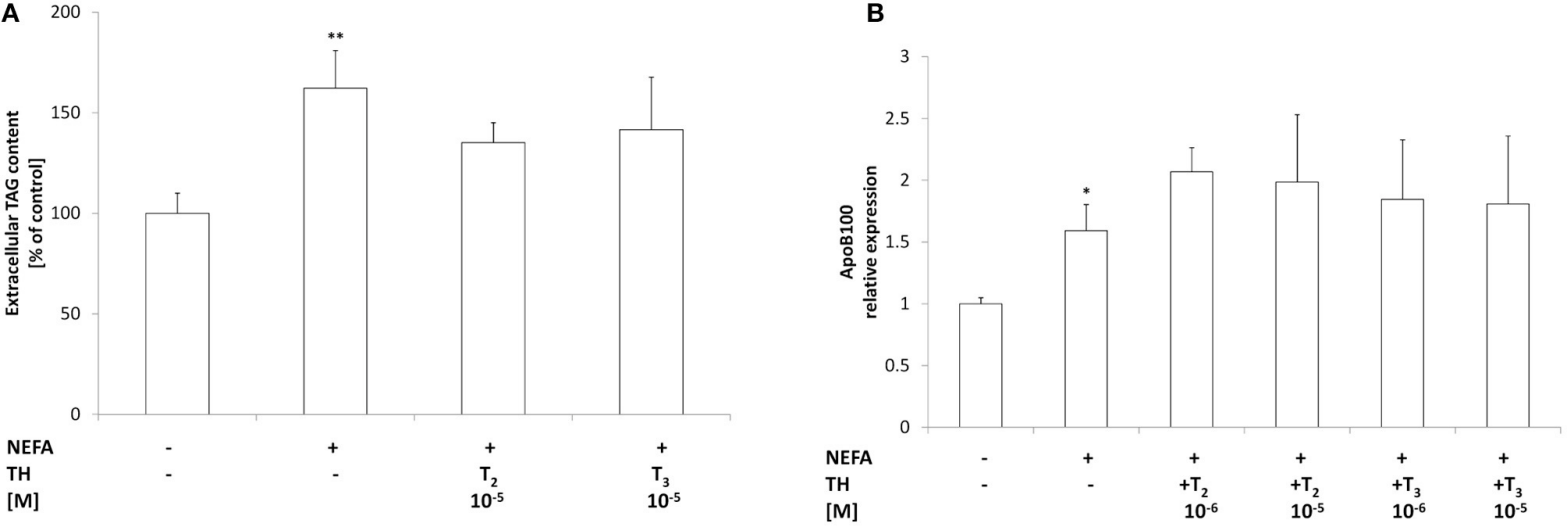
**Effects of iodothyronines on triglyceride secretion**. **(A)** The extracellular TAG content was quantified in the culture medium in control and “steatotic” cells incubated in the absence (NEFA) or in the presence of T_2_ or T_3_ (10^−5^M, 24 h). The mRNA **(B)** levels of ApoB 100 were measured by qPCR, in control and “steatotic” cells incubated in the absence (NEFA) or in the presence of T_2_ or T_3_ (10^−5^ M, 24 h). GAPDH was used as the internal control for quantifying gene expression. Data, expressed with respect to controls, are the mean ± S.D. of at least four experiments in triplicate. Significant differences are denoted by symbols on bars (C vs. NEFA ^*^p ≤ 0.05, ^**^p ≤ 0.01).

Total TAGs in the culture medium were quantified as a measure of lipid secretion (Figure 5A). Excess lipid accumulation in “steatotic” hepatocytes was associated with an increased TAG content in the medium (+60%;*p* ≤ 0.01 with respect to controls). Treatment of “steatotic” cells with either T_2_ or T_3_ (10^−5^ M) did not induce any change in extracellular TAG content with respect to'steatotic' cells.

Expression of ApoB100, the main component of VLDL, was also investigated (Figure 5B). A slight increase in ApoB100 mRNA expression was observed in'steatotic' hepatocytes (1.6 folds *p* ≤ 0.05 with respect to controls). Incubation of “steatotic” cells with either T_2_ or T_3_ (10^−5^ M) did not induce any change in ApoB100 expression with respect to NEFA-treated cells. Neither T_2_ nor T_3_ affected ApoB100 expression or TAG secretion in control cells (data not shown).

## Discussion

Thyroid hormones exert pleiotropic effects on the entire organism with a major role in modulating energy balance and lipid metabolism. Previous studies by us (Grasselli et al., 2008) and others (Lanni et al., 2005; Mollica et al., 2009) demonstrated the ability of thyroid hormones in reducing lipid accumulation in the liver. The direct effects of iodothyronines on hepatocytes have been investigated by developing an *in vitro* model of hepatic steatosis consisting of primary rat hepatocytes exposed to a mixture of oleate/palmitate (Grasselli et al., 2011a). In the present work, we demonstrate that the direct action of iodothyronines in reducing lipid accumulation in hepatocytes is due to an increased TAG mobilization from LDs likely mediated by ATGL recruitment on LD surface. Moreover, our data suggest that the excess FFAs deriving from ATGL action are likely addressed to mitochondria for beta-oxidation rather than to secretion as VLDL. Interestingly, despite the stimulation of lipid catabolic pathways, the lipid-lowering effect of iodothyronines is not associated with an increased rate of ROS production.

The iodothyronine-driven lipid-lowering action led to a reduction in total TAG content that parallels with the decrease in both number and size of LDs, as previously documented (Grasselli et al., 2011a). The effects of iodothyronines on LD size might be of some significance. Indeed, large LDs provide more efficient fat storage, whereas smaller LDs, with higher surface/volume ratio, facilitate the release of their stored lipids given the extensive surface accessible to lipases (Yu et al., 2015). Among the different lipases, ATGL is now universally recognized as the first and key enzyme in TAG hydrolysis in both adipose and non-adipose tissues (Watt and Steinberg, 2008). In this work, we show that the lipid-lowering effects exerted by iodothyronines *in vitro* can be ascribed, at least partially, to ATGL recruitment at the surface of LDs. In fact, in “steatotic” hepatocytes ATGL-positive droplets are rare and small, whereas numerous and large ATGL-negative LDs are present. Treatment of “steatotic” hepatocytes with iodothyronine stimulates both ATGL expression and recruitment at LD surface, suggesting an increased hydrolysis rate of LD-stored TAGs.

The dynamic nature of LDs is now recognized and ascribed to a proteomic equipment that varies depending on the metabolic status of the cell and, ultimately, of the entire organism (Crunk et al., 2013). In this context, we measured the expression of four LD-associated proteins: Rab18, TIP47, ADRP, and OXPAT. The latter did not change for all treatments tested. On the other hand, in “steatotic” hepatocytes expression of Rab18 and ADRP was induced. Treatment with iodothyronines induced a decrease in the expression of both Rab18 and TIP47. Rab18 is a LD-coating GTPase, whose role in lipid metabolism is still under debate, but it seems to be involved in basal lipogenesis and TAG accumulation (Kiss and Nilsson, 2014). Recently, several Rab proteins have been localized to LD surface, and some works revealed that GTP is a key mediator of LD-mitochondria interaction. In fact, a biophysical study identified protein–protein contacts between the surface proteins of these two organelles in yeast (Pu et al., 2011). TIP47 and ADRP seem to exert an overlapping action on LD formation and TAG synthesis as well as in protecting TAG from lipolysis (Sztalryd et al., 2006). Moreover, absence/reduction of TIP47 at the LD surface could facilitate the access of endogenous lipases, such as ATGL, to the stored TAGs (Bell et al., 2008). Although our data cannot demonstrate that the lipid-lowering effect of iodothyronines is directly due to modulation of Rab18 and TIP47 expression, this hypothesis cannot be discharged.

NEFAs released from LDs as a consequence of ATGL activity are available as substrates for subsequent oxidation (Reid et al., 2008). Fatty acid oxidation is increased by ATGL overexpression and decreased by ATGL knockdown (Ong et al., 2011). In this work, the stimulation of mitochondrial beta-oxidation exerted by iodothyronines in “steatotic” hepatocytes was suggested by two findings: *(i)* enhanced expression of CPT1 in order to increase entering of NEFAs into mitochondria; *(ii)* stimulation of COX enzymatic activity, the last enzyme in the respiratory electron transport chain of mitochondria. On the other hand, iodothyronine treatment did not alter the number of mitochondria (data not shown), thus indicating that the observed increase in COX activity *in vitro* can be ascribed to a direct action of iodothyronines on protein level and/or catalytic activity of this enzyme. This findings are in line with previous works demonstrating that the *in vivo* lipid-lowering action of T_2_ is due to mitochondrial beta-oxidation stimulation (Lanni et al., 2005; de Lange et al., 2011). Moreover, *in vitro*, a marked increase in CPT1 expression upon iodothyronine treatment was observed in FaO rat hepatoma cells, a model of rat hepatocytes defective for thyroid hormone receptors (TR) (Grasselli et al., 2011b), and this suggests that iodothyronines might exert their action on mitochondria through both TR-dependent and non-TR–dependent pathways converging on CTP1 expression. We wish to underline that another site for NEFA catabolism resides in peroxisomes, the main site for beta-oxidation of long- and very long-chain NEFAs (Musso et al., 2009). We previously showed that excess lipid accumulation is associated with an increased activity of acyl CoA oxidase (AOX), the key enzyme in peroxisomal oxidation of NEFA, but that iodothyronines decreased AOX activity (Grasselli et al., 2011a). Taken together our data indicate that the lipid-lowering effect of iodothyronines is likely due to a stimulation of mitochondrial rather than peroxisomal oxidation.

Generation of ROS by active mitochondria is very well known as a cause of increased oxidative stress (Boveris and Chance, 1973). Our *in vitro* model of hepatic steatosis is not associated to oxidative stress, since no changes were observed neither in TBARS level nor in the expression of antioxidant molecules such as MT-1 and MT-2. Even iodothyronine treatment of steatotic hepatocytes did not rise TBARS level, indicating that neither T_3_ nor T_2_ altered the oxidative homeostasis of the cell. The finding that both T_2_ and T_3_ at higher concentration induced a decrease in the mRNA levels of both MT-1 and MT-2 isoforms is in accordance with previous data form ours demonstrating a marked decrease in the activities of superoxide dismutase and catalase when “steatotic” hepatocytes were incubated with iodothyronines (Grasselli et al., 2011a).

NEFAs released from LDs can be subjected to another pathway and can be readdressed to ER, re-esterified, packaged and secreted as VLDL. Increasing VLDL secretion can be a compensatory mechanism in fatty liver. In our *in vitro* model, excess lipid accumulation induced an increase in ApoB100 expression and in the concentration of extracellular TAG, thus indicating that “steatotic” hepatocytes try to overcome lipid overload by increasing TAG secretion rate. On the other hand, iodothyronines did not influence VLDL secretion or ApoB100 expression when administered to steatotic cells. Of note, these data may be strictly dependent on the doses and the duration of the used hormonal treatment, as well as on the *in vitro* experimental conditions. Indeed, some recent *in vivo* data have furnished different evidences: when administered to western type diet fed low-density lipoprotein (LDL) receptors knockout mice, thyroid hormones dramatically reduce circulating total and VLDL/LDL cholesterol and this cholesterol reduction is associated with decreased circulating levels of both ApoB48 and ApoB100 (Goldberg et al., 2012).

Taken together, our data indicate that both T_2_ and T_3_ reduce the fat content in “steatotic” hepatocytes by triggering ATGL recruitment on LD surface and stimulating mitochondrial beta-oxidation rather than TAG secretion and peroxisomal oxidation. This is in accordance with previous report indicating that ATGL knockdown is associated with decreased mitochondrial fatty acid oxidation without any effects on TAG secretion (Ong et al., 2011). In this scenario, ATGL could be considered a mediator of the lipid-lowering action of iodothyronines on hepatocytes by channeling hydrolyzed NEFA toward mitochondrial beta-oxidation.

## Author contributions

All authors contributed to this work significantly. EG carried on the planning of experiments, performed cell treatments, elaborated the data and drafted the manuscript; ID performed the hepatocyte isolation and culture and contributed to manuscript writing; AV participated in conceiving and designing the study and revised the manuscript; AC and GV carried out experiments of quantitative RT-PCR and TBARS quantification; RD performed ATGL-immunohistochemical analyses; FG and ES designed and performed analyses of COX in-gel activity; GG gave a contribution in study design, revised the manuscript and financially supported this work; LV conceived and designed the study, supervised the experimental activities and data elaboration, wrote the manuscript.

## Funding

Contract grant sponsor: MIUR-COFIN (Prot. 20089SRS2X_002), Compagnia San Paolo Torino, Fondi Ateneo Università degli Studi di Genova, Area-05 Scienze Biologiche, and Fondazione CARIGE.

### Conflict of interest statement

The authors declare that the research was conducted in the absence of any commercial or financial relationships that could be construed as a potential conflict of interest.
